# Effect of Immuno-Nutrition on Malnutrition, Inflammatory Response and Clinical Course of Semi-Critically Ill COVID-19 Patients: A Pilot Perspective Study

**DOI:** 10.3390/nu15051250

**Published:** 2023-03-02

**Authors:** Marialaura Scarcella, Emidio Scarpellini, Sara Piergallini, Emanuele Rinninella, Karen Routhiaux, Carlo Rasetti, Ludovico Abenavoli, Edoardo De Robertis, Pietro Manzi, Rita Commissari, Riccardo Monti, Michela Zanetti

**Affiliations:** 1 Anesthesia, Intensive Care and Nutritional Science—Azienda Ospedaliera “Santa Maria”, Via Tristano di Joannuccio, 05100 Terni, Italy; 2 Clinical Nutrition Unit and Internal Medicine Unit, “Madonna del Soccorso” General Hospital, Via Luciano Manara 7, 63074 San Benedetto del Tronto, Italy; 3 Translational Research Center for Gastrointestinal Disease (T.A.R.G.I.D.), Gasthuisberg University Hospital, KULeuven, Herestraat 49, 3000 Lueven, Belgium; 4 School of Nursing, Politechnics University of Marche, 60121 Ancona, Italy; 5 Department of Translational Medicine and Surgery, Clinical Nutrition Unit, Catholic University of Sacred Heart, Gemelli Foundation, 00168 Rome, Italy; 6 Department of Health Sciences, University “Magna Graecia”, 88100 Catanzaro, Italy; 7 Division of Anaesthesia, Analgesia, and Intensive Care - Department of Medicine and Surgery-University of Perugia, 06121 Perugia, Italy; 8 Azienda Ospedaliera Santa Maria di Terni, 05100 Terni, Italy; 9 Anesthesia and Intensive Care Azienda Ospedaliera Santa Maria Terni, 05100 Terni, Italy; 10 Cardiologic and Pediatric Intensive Care Unit, 05100 Terni, Italy; 11 Department of Medical and Surgical Sciences, Azienda Sanitaria Universitaria “Giuliano-Isontina”, Trieste University, 34148 Trieste, Italy

**Keywords:** COVID-19, malnutrition, over-weight, immune-nutrition, inflammation, semi-intensive care

## Abstract

Background: The SARS-COV 2 pandemic has hit on our lives since early 2020. During different contagion waves, both malnutrition and overweight significantly correlated with patient mortality. Immune-nutrition (IN) has shown promising results in the clinical course of pediatric inflammatory bowel disease (IBD) and in both the rate of extubation and mortality of patients admitted to an intensive care unit (ICU). Thus, we wanted to assess the effects of IN on a clinical course of patients admitted to a semi-intensive COVID-19 Unit during the fourth wave of contagion that occurred at the end of 2021. Methods: we prospectively enrolled patients admitted to the semi-intensive COVID-19 Unit of San Benedetto General hospital. All patients had a biochemical, anthropometric, high-resolution tomography chest scan (HRCT) and complete nutritional assessments at the time of admission, after oral administration of immune-nutrition (IN) formula, and at 15 days interval follow-up. Results: we enrolled 34 consecutive patients (age 70.3 ± 5.4 years, 6 F, BMI 27.0 ± 0.5 kg/m^2^). Main comorbidities were diabetes (20%, type 2 90 %), hyperuricemia (15%), hypertension (38%), chronic ischemic heart disease (8 %), COPD (8%), anxiety syndrome (5%), and depression (5%). 58% of patients were affected as moderately-to-severely overweight; mini nutritional assessment (MNA) score (4.8 ± 0.7) and phase angle (PA) values (3.8 ± 0.5) suggestive of malnutrition were present in 15% of patients, mainly with a history of cancer. After 15 days upon admission, we recorded 3 deaths (mean age 75.7 ± 5.1 years, BMI 26.3 ± 0.7 kg/m^2^) and 4 patients were admitted to the ICU. Following IN formula administration, inflammatory markers significantly decreased (*p* < 0.05) while BMI and PA did not worsen. These latter findings were not observed in a historical control group that did not receive IN. Only one patient needed protein-rich formula administration. Conclusions: in this overweight COVID-19 population immune-nutrition prevented malnutrition development with a significant decrease of inflammatory markers.

## 1. Introduction

From January 2020, the novel corona virus (SARS-CoV2) disease, firstly believed to be characterized by pneumonia only (namely, COVID-19), spread around the world. A terrible pandemic has caused healthcare systems to crash with a high damage in terms of lives and morbidity to humanity. Wave after wave of COVID-19 pandemic helped researchers to understand and assemble a clearer and clearer knowledge of this hyper-inflammatory syndrome. In fact, it was true that a significant percentage of patients developed a serious bilateral pneumonia, resembling severe acute respiratory syndrome (SARS) and Middle East respiratory syndrome (MERS), but other symptoms and clinical manifestations were prevalent also. For example, several patients were developing pulmonary thrombo-embolism, encephalitis, and hemorrhage [1]. Interestingly, COVID-19 pneumonia is often associated with gastrointestinal disorders that do not allow the patient to be adequately fed, especially in the pre-Intensive Care Unit (ICU) stages [1,2].

Although the severity of the clinical condition has been reported to be milder than SARS with a mortality rate ranging from 4.3 to 11%, the latter has been a huge wound for our societies [2]. In detail, there is a direct correlation between inflammatory status, incidence of comorbidities, and mortality in COVID-19 patients [2]. Looking at physiology, main defensive mechanisms in the human body against viruses, in general, and SARS-CoV2, specifically, are the physical barriers (namely, skin, and mucosal membranes): stomach acid content and digestive enzymes, gut microbiome, and innate and acquired immunity [2]. Many of the reactions maintaining these mechanisms need vitamins A, D, B, iron, and zinc as coenzymes. This is one of the main reasons why setting an appropriate nutritional strategy as a part of the treatment is crucial for survival of these patients [2].

There is a clear correlation between obesity and mortality in COVID-19 patients. In fact, sarcopenia as feature of malnutrition, typical of obesity, is significantly associated with micro-inflammation and major allowance of SARS-CoV 2 entrance into target cells [3]. 

To date, there are limited therapeutic remedies available for the treatment of COVID-19. Thus, nutritional modulation of the immune system function has been investigated both as a preventive and curative option [4,5]. In fact, there is a growing number of recently published key studies suggesting promising effects of immuno-nutrition on acute respiratory infections [6,7]. Indeed, immuno-nutrition can be defined as “modulation of either the activity of the immune system or modulation of the consequences of activation of the immune system by nutrients or specific food items fed in amounts above those normally encountered in the diet” [8]. Recently, specific immuno-nutrients have been proposed as effective items for both or add-on treatments of COVID-19 vs. evidence-based standard therapy, promising results for the reduction of innate and adaptive immune response responsible of “cytokines’ storm” typical of COVID-19 patients [9].

In ICU patients, the inflammatory status is associated with an increased mortality [3,4]. For example, the higher is the Nutritional Risk Score (NRS), the higher the incidence of acquired healthcare-associated infections and the mortality risk index [3,5]. 

The ICU COVID-19 patient is a frail one with multiple comorbidities, affected by hypoxia, inflammation, high body temperature, increased oxygen demand, and often prone to malnutrition. In the early stages of COVID-19, patients experience anorexia exacerbated by severe coughing, fever, dyspnea, anosmia, hypoxia, and fatigue, causing difficulties to maintain an appropriate nutritional oral intake. Moreover, before ICU admission, the patients are often treated with Continuous Positive Airway Pressure (CPAP) or Non-invasive Ventilation (NIV) that do not allow oral feeding in a large percentage.

Previous data from our study group demonstrated the efficacy of whey-protein-rich enteral feeding formula in ICU ventilated COVID-19 patients with earlier extubation time and improved nutritional status [10].

Thus, this prospective observational exploratory single-center study aims to evaluate the nutritional and anti-inflammatory effects of an outlined nutritional protocol based on immuno-nutrition (IN) in COVID-19 patients admitted to the semi-intensive COVID-19 unit. 

## 2. Materials and Methods

### 2.1. Study Protocol

In this single-center perspective exploratory study, we consecutively enrolled COVID-19 adult patients admitted at the semi-intensive Unit of “Madonna del Soccorso“ General Hospital, San Benedetto del Tronto, Italy between 1 September and 31 December 2021. We respected regional Ethical Committee rules for patients’ enrollment (Ethical Committee Marche, Italy). Inclusion criteria were: age > 18 years, confirmed diagnosis of SARS-CoV2 infection, and need for non-invasive mechanical ventilation for at least 48 h. Patients were treated according to the updated guidelines for COVID-19 [11].

All patients had a biochemical, anthropometric, high-resolution tomography chest scan (HRCT) and complete nutritional assessments (MNA test and bioimpedance vector analysis (BIVA)) at the time of admission and at 15 days interval follow-up, namely after daily oral administration of immune-nutrition (IN) formula.

The study group was compared with historical COVID-19 patients not administered with IN formula.

### 2.2. Inclusion and Exclusion Criteria

We included consecutive patients admitted to the semi-intensive Unit of San Benedetto General Hospital in need of NIV because of SARS-CoV 2 infection.

Exclusion criteria were: pregnancy, artificial nutrition in the previous 15 days upon admission, allergy to the immuno-nutrition components, major GI tract surgery, malapsorption syndromes, inflammatory bowel disease, GI motility disorders, acute or chronic pancreatitis, immudepression (e.g., acquired immunodepression syndrome (HIV)), hematologic disease, and cognitive status impairment. 

### 2.3. Immune-Nutrition Administration Scheme

The immune-unutrition (IN) formula used in the study is a powdered oral nutritional supplement designed for patients affected by inflammatory bowel disease. In fact, there is much evidence confirming its anti-inflammatory effect, especially in inflammatory bowel disease in children [12].

Its composition consists of: proteins 3.5 g/100 mL (consisting exclusively of casein naturally rich in TGF-ß2); fats 4.6 g/100 mL (milk fat, MCT, corn oil, soy lecithin. MCT: 25% of total lipids, in order to facilitate rapid replenishment; essential fatty acids equivalent to 4.6% of total calories; limited content of linoleic acid (n-6)); carbohydrates 11 g/100 mL (maltodextrin (61%) and sucrose (39%).

The powder is reconstituted at 20%—1 Kcal/mL: 200 g of powder in 850 mL of water, to re-constitute 1 L of IN formula (1000 Kcal). Later, it is possible to increase the concentration up to 30%-1.5 Kcal/mL: 300 g of powder in 750 mL of water to reconstitute 1 L (1500 Kcal) [12].

The formula was administered once daily (in detail, 300 g of powder in 750 mL of water to reconstitute 1 L (1500 Kcal)) together with the diet of the patient, delivering on average 30–40% of the total calories of the daily diet.

### 2.4. Nutritional Assessment

#### 2.4.1. Mini Nutritional Assessment (MNA) Test

The Mini Nutritional Assessment is a multidimensional screening tool, validated in many clinical settings. More specifically, it is an integrated nutrition index that evaluates different nutritional parameters in order “to obtain a synthetic information and a more accurate nutritional diagnosis” [13]. MNA has 96% sensitivity, 98% specificity, and 97% predictive value to describe nutritional status of patients [14]. Moreover, MNA is easily repeatable and can be used also by non-trained nutritionists [2].

MNA can be used both as a first-level screening and for follow-ups in elderly patients [15]. Interestingly, in hospitalized elderly patients, MNA scores can help predict healthcare costs, length of stay, and short-term and long-term mortality. In fact, MNA test shows an inverse correlation with these variables [16,17]. 

MNA test is a reliable index of muscle disability and motility and, also a complementary tool for nutritional status assessment in patients [18].

The MNA test is composed by 18 items divided into three sections: one for anthropometry and weight changes; one that evaluates quality and quantity of food intake; one measuring disabilities and cognitive status [19].

There are two steps:

- Screening (maximum score of 14 out of six variables): story of weight loss in the previous three months, food intake, motility, acute stress, cognitive status, and Body Mass Index (BMI) assessment. In particular, score of 0–7 is predictive of malnutrition, a score of 8–11 suggests that patients are at risk of malnutrition, and a score of 12–14 indicates that the person is well nourished and needs no further investigation. If the score is less than 11 it is strongly recommended to continue with the remaining test items.A MNA score higher than 24 indicates the patient is well-nourished, a score between 17–23.5 suggests a risk of malnutrition and scores lower than 17 clearly highlight malnutrition.
- Self-Global Assessment (history of drugs assumption, food habits, fluid intake, residence place, and patient’s considerations on personal health status and on nutritional status).

#### 2.4.2. Bioimpedance Vector Analysis 

Bioelectrical impedance analysis (BIA) is a non-invasive tool to assess human body composition (i.e., analysis of fat, bone, water, and muscle content). BIA delivers a low frequency electrical current and is based on the principle that fluid and cellular structures present different levels of resistance to an electrical current when it passes through a living system [20]. In particular, BIA measures: Resistance (R-Ohms), assessing cellular hydration; Reactance (Xc—Ohms), assessing tissue integrity and Phase Angle (PA—degrees), representing the arc tangent between R and Xc. Thus, BIA serves to evaluate hydration and nutrition in humans [21].

Bioelectrical impedance vector analysis (BIVA) assesses body composition in advanced illness such as intensive care admitted patients. In fact, statistical vector analysis of BIA data leads to human body composition measurements in this particular subset of patients [22]. Bioelectrical impedance vector analysis (BIVA) is made with graphical vectors to analyze BIA data. Thus, impedance (Z) is plotted as a vector from its components R (*X*-axis) and Xc (*Y*-axis), after being standardized by height (H). The RXc graph represents the sex- and race-specific tolerance intervals of a comparative reference population. Tolerance ellipses are plotted on the RXc graph to represent the 50%, 75%, and 95% centiles (i.e., confidence intervals) for the population in study. This method allows a simultaneous assessment of changes in tissue hydration or soft tissue mass, independent of regression equations, or body weight. For these reasons, BIVA can be interpreted accurately also in critically ill ICU patients that are at extremes of weight or volume distribution. 

### 2.5. Data Collection 

We prospectively collected antropometric, clinical and laboratory tests’ data from the patient’s medical file. In detail, we collected general and demographic variables on the day of semi-intensive unit admission. All the other data and parameters measured were recorded daily for the entire patients’ stays, starting from admission to discharge/death. In particular, we recorded inflammation and infection markers (CRP, IL-6, white blood cells count and formula, procalcitonin, and erythrocyte sedimentation rate), renal and hepatic function indices, and blood gas analysis variables. The collected data were filled in a database guaranteeing the anonymity of the patients.

### 2.6. Statistical Analysis 

Statistical analysis was performed with SPSS Software 21 (IBM, New York, NY, USA). Preliminarily, quantitative variables’ distribution was assessed with the Kolmogorov-Smirnov normality test. All data are presented as mean ± standard deviation (SD) or median [interquartile range, IQR] according to the normal or not normal distribution. Parametric (Student’s *t*-test) and non-parametric tests (Mann-Whitney U test) were applied to describe the differences between groups for the variables of interest, when appropriate. The alpha level of significance was set at 0.05 [23].

## 3. Results

From 1 September and 31 December 2021, we consecutively enrolled 34 COVID-19 adult patients admitted at the semi-intensive Unit of “Madonna del Soccorso” General Hospital, San Benedetto del Tronto, Italy. 

Mean age of the she study population (namely, COVID-19 IN) was 70.3 ± 5.4 years, 5 females, BMI 27.0 ± 0.5 kg/m^2^. 

Main comorbidities were diabetes (20%, type 2 90 %), hyperuricemia (15%), hypertension (38%), chronic ischemic heart disease (8%), chronic obstructive pulmonary disease (COPD) (8%), anxiety (5%), and depression (5%).

Considering inflammatory markers at enrollment, median CRP was 19 [5.6–31] mg/L; IL-6 101 pg/mL; white blood cells count 8070 (6263–11,000). 

HRCT scan results were as following: mild pneumonitis (30%), moderate pulmonary parenchima involvement (45%), and severe involvement (25%). 

Control group (*n* = 20) (COVID-19 patients not giving informed consent to IN) characteristics shown in Table 1. 

**Figure 1:**
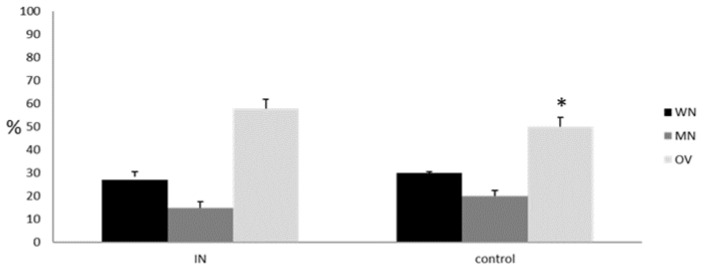
Nutritional status according to MNA test and BIVA assessment in IN and control group; WN: well-nourished; MN: malnourished; OV: overweight; * *p* < 0.05.

Comorbidities prevalence (data not shown) and other antropometric, nutritional, and inflammatory characteristics were comparable except for female sex representation and BMI (*p* = 0.05). In addition, MNA test results and BIVA confirmed a statistical difference for overweight representation between study and control group (both, *p* < 0.05) (Figure 1).

During semi-intensive unit stay all IN and control group patients were treated with guidelines-guided treatments (namely, remdesevir, metilprednisolone, piperacillin/tazobactam, and levofloxacin). There was no statistical difference among groups for medications used (*p* = NS). There was no difference on non-invasive mechanic ventilation type duration used among groups (*p* = NS).

**Figure 2:**
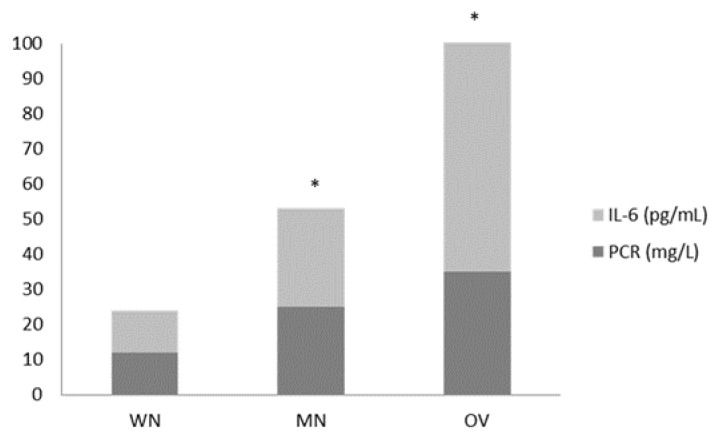
CRP and IL-6 values according to nutritional status (WN: well-nourished; MN: malnourished; OV: overweight) in the IN group at T0. * *p* < 0.05.

Figure 2 shows inflammatory markers values in IN group according to their nutritional status at T0. Control group showed a similar behavior (data not shown) at T0. In both groups, malnutrition and overweight were significantly associated with higher CRP and IL-6 values (both, *p* < 0.05). 

After 15 days of semi-intensive unit stay (namely T1), we observed 3 deaths (mean age 75.7 ± 5.1, 1F, BMI 26.3 kg/m^2^) and two patients were moved to ICU care in the IN group because of respiratory performance worsening. The latter was associated with worsened HRCT pneumonitis findings. 

In the control group, at T1 we observed 2 deaths (mean age 70.1 ± 3.1, 1F, BMI 23.5 kg/m^2^) and two patients were moved to ICU care because of respiratory performance worsening. The latter was associated with worsened HRCT pneumonitis findings. 

**Figure 3:**
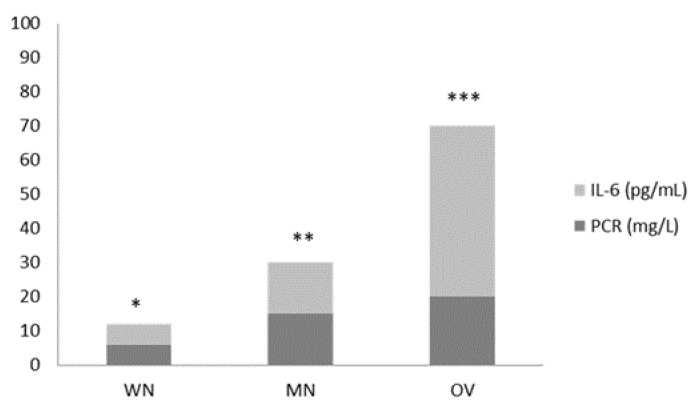
Reduction of inflammatory response markers (IL-6 and CRP) according to nutritional status in the IN group (T0 vs. T1; *, **, ***, all *p* < 0.05). A similar tendency was observed in the control group, without reaching statistical significance.

After 2 weeks of IN formula administration, we observed a significant reduction of inflammatory markers (PCR, IL-6), for both *, **, *** *p* < 0.05 in the IN group (Figure 3). In the control group, a similar trend was observed, without reaching statistical significance (*p* = NS) (data not shown). Glycemic assessment was not affected by IN nutrition (data not shown, *p* = NS).

**Figure 4:**
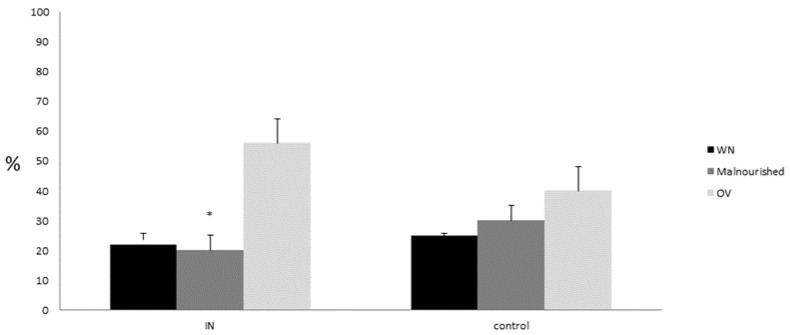
T1 nutritional status changes in the IN and control group. Malnutrition prevalence was significantly higher in the control group vs. IN treated-one. * *p* < 0.05.

Figure 4 describes nutritional status change in the IN and control group at T1. Immuno-nutrition administration was able to prevent nutritional status worsening in the treatment vs. control group (* *p* < 0.05).

Semi-intensive unit days of stay were not affected by IN use (*p* = NS).

## 4. Discussion

In this single-center perspective pilot study, COVID-19 patients admitted to a semi-intensive unit of our hospital were evaluated for the impact of immuno-nutrition on nutritional status and inflammatory response vs. a historical control group of COVID-19 patients not administered with IN formula. 

We have shown, for the first time, that immuno-nutrition is able to prevent worsening of nutritional status in COVID-19 patients with a consensual inflammatory response reduction. A similar trend was observed for inflammatory markers only, as well as in the control group. 

These findings are in line with the previous report from our study group, although in those investigation, patients were administered with whey protein-rich formula and treated in the ICU ward [10]. The finding of prevention of malnutrition development observed in the present study can be explained by an accurate nutritional assessment operated in these semi-intensive patients in our secondary care center. In fact, COVID-19 patients are difficult to assess because of difficulties related to individual protective disposables use. Only a fine organization allows health care operators to prevent malnutrition development in SARS-CoV2 patients, improving their respiratory performance and reducing their morbidity and mortality [10]. 

In the present study there was not a significant correlation between prevention of malnutrition development and improved mortality or prevention of worsened clinical course (namely, need for ICU admission). This finding can be explained by the small sample size and short follow-up time of the population in study that do not allow further speculation on the impact of IN administration on prognosis of COVID-19 patients. 

There is solid evidence showing the positive impact of nutritional assessment and use of specific protein-rich food nutrient supplement on COVID-19 patients’ morbidity and mortality [24]. In detail, in the literature there are reports that evaluated the impact of nutrition in ICU COVID-19 patients with early manifestations of malnutrition and, sarcopenia [25]. Both of these conditions are significantly associated with morbidity and mortality rate of critical and rehabilitation patients [20,26]. 

In this study we explored the impact of a formula rich in casein used with success in pediatric IBD populations [12]. We hypothesized that this formula with IN properties was able to reproduce effects described in gastrointestinal tract inflammatory conditions such as Chron disease and ulcerative colitis. In fact, COVID-19 is characterized by hyper-inflammatory state. Thus, we went over the study of the impact of adequate nutritional status care in COVID-19 patients. 

In fact, several reports from literature have shown how the use of pre-, pro-, and postbiotics is able to be efficient as add-on treatment for steroids, antibiotics, and antivirals against the entrance of SARS-CoV2 into our body cells [27,28]. Moreover, these remedies can help reducing the cytokines’ storm typical of COVID-19 [29]. However, some data are available on immuno-nutrition in ICU and non-ICU patients, respectively.

To date, we found only one report from Brazil evaluating the impact of hyper-proteic normo-caloric diet with or without IN formula add-on on the inflammatory response and related lymphopenia in non-ventilated COVID-19 patients [30]. On the other hand, our investigation evaluated patients under non-invasive ventilation in the semi-intensive ward. Thus, these patients had a higher inflammatory response and more severe pulmonary involvement than the study by Pimentel et al. Moreover, the other investigation did not report significant effects of IN formula administration on nutritional status. 

Although in the control group a similar trend was observed for the reduction of inflammatory markers, only the group of patients treated with IN demonstrated a statistically significant reduction of IL-6 and CRP. Thus, these findings support an anti-inflammatory effect of IN. In particular, omega-3 fatty acids are essential in the prevention and treatment of cardiovascular and auto-inflammatory disease [31,32]. 

For example, both diabetic and septic patients showed a reduction of CRP and other inflammatory cytokines after ω -3 fatty acids administration (another IN formula). Specifically, in a 2020 trial performed in Iran, 128 COVID-19 ICU patients were randomized to standard diet with/without add-on of ω -3 fatty acids. Compared with those who did not receive omega-3 fatty acids, treated patients presented a significant improvement of renal function and a reduction of systemic inflammatory response. This anti-inflammatory effect can be explained by the “competition” between fatty acids and SARS-CoV-2 for cell entrance. In fact, ω -3 fatty acids can bind viral spike protein and modify its spatial conformation, resulting in a lower viral load for the infected host [33].

In another study from France, 26 COVID-19 patients admitted to the ICU ward showed a significantly higher myeloid-derived suppressor (MDSC) cell activation, associated with the typical COVID-19-driven lymphopenia. After administration of arginine, these patients showed a significant reversal of lymphopenia. However, the sample size of study was too small to drive definitive conclusions [34].

More in detail, arginine seems to be able to reduce SARS-CoV-2 infectivity. Further evidence on this capability is derived from molecular biology data: isoleucine replacement with arginine in the 407 position of spike protein worsen its interaction with the human Angiotensin-Converting Enzyme 2 (ACE2). The latter is crucial for the virus cell infection. 

The IN formula used in this study has particular characteristics. It has been successfully used in pediatric inflammatory bowel disease (IBD) patients [12]. In fact, the add-on use of bioactive peptides to the industrial diet may favor mucosal healing in Crohn disease (CD) patients because of their anti-inflammatory effect [12,35]. In detail, bioactive peptides are specific growth factors such as transforming growth factor-β (TGF-β). The latter belongs to the group of multifunctional regulatory peptides produced by various cell types. Particularly, TGF-β controls the processes of lymphocytes, macrophages, and dendritic cells differentiation, proliferation, and activation. Thus, TGF-β has a strong anti-inflammatory effect and can prevent the development of autoimmune diseases [34]. In CD, in particular, and IBD patients, in general, inflammation reduction obtained through this IN formula administration has been assessed endoscopically and by fecal calprotectin (FC) dosage [12,34]. Thus, local immunomodulation has been confirmed in this subset of patients. In our study, systemic hyper-inflammation state is present and IN administration can help in down-regulating this process (e.g., as expressed by the significant decrease of CRP and IL-6). However, more data are needed to confirm this preliminary finding, perhaps with the dosage of FC also. 

Our study has several limitations. First, the sample size was small, according to the pilot design of the study. Second, our study took into consideration the third wave of the SARS-CoV 2 pandemic where virus strain, vaccine, and antivirals use had changed the clinical and laboratory characteristics of the disease. Third, our cohort in treatment had a low representation of female sex and high representation of obese people. The latter could have conditioned the prevalence of sarcopenia, and therefore have conditioned the results. 

## 5. Conclusions

In conclusion, data from this pilot single-center perspective study showed that immuno-nutrition is able to prevent malnutrition development in COVID-19 patients admitted in semi-intensive unit, together with a significant reduction of pro-inflammatory cytokines’ storm. The potential relationship between risk of malnutrition reduction and fall of hyper-inflammatory response in these patients needs to be further investigated. However, these promising results are conditioned by the small sample size of patients enrolled in a single-center secondary hospital. Thus, larger sample size and multi-centric randomized placebo-controlled studies are needed to confirm these results. 

## Figures and Tables

**Table 1:** Characteristics of the study and control populations at T0.

	IN COVID-19 pts (n = 34)	Control Group (n = 20)	*p*-Value
**Age (years)**	70.3 ± 5.4	68.0 ± 5.5	NS
**sex**	6 F	8 F	0.05
**BMI (kg/m^2^** **)**	27.0 ± 0.5	23.5 ± 0.6	<0.05
**MNA test (WN/MN/OV (** **%))**	27/15/58	30/22/48	<0.05
**PA (°** **) (** **WN/MN/OV** **)**	4.5/3.8/8.2	4.4/3.2/7.8	<0.05
**CRP (mg/**L**)**	19 (5.6–31)	20 (6–33)	NS
**IL-6 (pg/mL)**	101 (35–133)	103 (34–136)	NS
**S** **emi-intensive unit stay (days)**	18.2 ± 0.4	18.6 ± 0.6	NS

Table legend: NS: non-significant; F: female sex; MNA: mini nutritional assessment test; WN: well-nourished; MN: malnourished; OV: over-weight; PA: phase angle obtained through bioimpedance vector analysis (BIVA) measurement; CRP: C reactive protein; IL-6: interleukine 6.

## Data Availability

Data supporting these results can be found in the patients’ files database of “Madonna del Soccorso General Hospital”, San Benedetto del Tronto, Italy.
